# Bone mineral density changes during pregnancy in actively exercising women as measured by quantitative ultrasound

**DOI:** 10.1007/s00404-012-2315-5

**Published:** 2012-04-04

**Authors:** William W. K. To, Margaret W. N. Wong

**Affiliations:** 1 Department of Obstetrics and Gynaecology, United Christian Hospital, 130 Hip Wo Street, Kwun Tong, Kowloon, Hong Kong SAR, People’s Republic of China; 2 Department of Orthopaedics and Traumatology, Prince of Wales Hospital, Hong Kong SAR, People’s Republic of China

**Keywords:** Bone mineral density, Pregnancy, Exercising women

## Abstract

**Objective:**

To evaluate whether bone mineral density (BMD) changes in women engaged in active exercises during pregnancy would be different from non-exercising women.

**Methods:**

Consecutive patients with singleton pregnancies who were engaged in active exercise training during pregnancy were prospectively recruited over a period of 6 months. Quantitative USG measurements of the os calcis BMD were performed at 14–20 weeks and at 36–38 weeks. These patients were compared to a control cohort of non-exercising low-risk women.

**Results:**

A total of 24 physically active women undergoing active physical training of over 10 h per week at 20 weeks gestation and beyond (mean 13.1 h, SD 3.3) were compared to 94 non-exercising low-risk women. A marginal fall in BMD of 0.015 g/cm^2^ (SD 0.034) was demonstrable from early to late gestation in the exercising women, which was significantly lower than that of non-exercising women (0.041 g/cm^2^; SD 0.042; *p* = 0.005). Logistic regression models confirmed that active exercises in pregnancy were significantly associated with the absence of or less BMD loss in pregnancy.

**Conclusion:**

In women actively engaged in physical training during pregnancy, the physiological fall in BMD during pregnancy was apparently less compared to those who did not regularly exercise.

## Introduction

Various methods to assess the changes in bone mineral density (BMD) during pregnancy have been studied. The use of standard dual-energy X-ray absorptiometry (DXA) in pregnant women has been limited by the potential harmful effects of radiation during pregnancy. Studies that utilize this method for assessments would obtain measurement in women before pregnancy and then repeat it in the early postpartum period [1, 2], yet the actual changes during pregnancy could not be assessed. The recent development in quantitative ultrasound methods for assessment of BMD in pregnancy carries the particular advantage of being free from irradiation effects [3–8]. Ultrasound measurements have been found to correlate well with BMD measurements compared to conventional DXA methods in non-pregnant subjects, and could be used alone for prediction of fracture risks in postmenopausal women [9]. Ultrasound measurements may be performed at different sites, including the tibia [3–5], os calcis [6, 10], metacarpals and phalanges [7, 8]. Serial USG measurements across advancing gestations in pregnancy have been able to show consistent progressive BMD loss in different bone sites. The degree of BMD changes during pregnancy has been correlated with higher bone turnover as indicated by biochemical markers [3], as well as with maternal characteristics such as low initial BMD in early pregnancy and high body fat accumulation during pregnancy [6]. It could also be related to calcium intake and mechanical stress or increased bone loading during pregnancy [10, 11].

The positive effect of exercises on BMD has been well supported in the literature. In pre-pubertal children and adolescents, physical training and high-impact exercises have been associated with higher BMD accrual [12, 13]. This effect was observed to continue into reproductive age women [14, 15]. There is also evidence that physical activity effectively slows bone loss in postmenopausal women in a dose-dependent manner [16]. This study aimed at comparing the longitudinal changes in BMD in a cohort of pregnant women who continued to engage in regular physical training till the third trimester with women who did not exercise using quantitative ultrasound measurement of the os calcis. Such comparison should help to define whether physical exercises during this very dynamic period of bone metabolism would positively reduce the normal physiological BMD loss in pregnancy.

## Methods

Consecutive patients with singleton pregnancies booked at a general obstetric clinic in a regional hospital were prospectively recruited for the study over a 6-month period. The obstetric department was a tertiary referral center in the region and part of a university teaching unit which catered to an annual delivery of around 5,000 women. A short screening questionnaire was administered to women booking before 20 weeks to obtain details of their occupation and physical exercise level. Those who were screened to be actively exercising were approached by the investigators individually to obtain a more elaborate history of their exercise details, including the specific types of sports or training engaged in, the intensity and time spent on the exercises per week, and whether such exercises were occupation related or performed at leisure. The inclusion criteria for the study included weight-bearing exercises performed regularly over 10 h per week (at least five 2-h days per week). Basic epidemiological data, including early pregnancy weight and height were recorded. Quantitative ultrasound bone density measurements were performed at the os calcis bilaterally between 14 and 20 weeks, and body fat percentage was estimated using bio-impedance methods. Routine antenatal care was offered in accordance with our service protocol. These recruited women were interviewed individually again in the third trimester between 32 and 36 weeks, and their level of exercise during pregnancy was again assessed. Quantitative ultrasound bone density measurements and body fat estimations were then repeated in the third trimester between 36 and 38 weeks.

A group of women screened negative for exercises were recruited during the same period as controls. To eliminate selection bias, by default, these controls were the four patients consecutively booked after the index patients. They were approached by the investigators individually and their exercise levels were re-confirmed. The eligible criteria for controls were women who had no regular job-related or leisure exercises for more than 2 h per week. Basic epidemiological data were recorded and quantitative ultrasound bone density and body fat measurements were performed at 14–20 weeks and at 36–38 weeks as in the study group. Patients with significant medical disorders during the antenatal period, including gestational hypertension, diabetes mellitus and those who delivered preterm before 37 weeks of gestation, were excluded from recruitment or analysis. The study was approved by the cluster hospital ethics committee and written consent was obtained from all participating women.

Quantitative ultrasound bone density measurements were done using the Sahara Clinical Bone Sonometer system (Hologic, MA, USA), a waterless portable system that involved direct contact of the probe with the heel through elastomer pads and oil-based coupling gel. The system was able to generate a simulated BMD value derived from the basic speed of sound and bone ultrasound attenuation parameters, and this was used in the subsequent analysis for calculations. Body fat percentage assay was also performed using a Tanita 500 bio-impedance system (Tanita, Tokyo, Japan). The system utilized a single frequency of 50 kHz to measure the resistance to flow of an electric current that was passed through body fluids and the percentage of body fat based on a two-compartment model of fat mass and lean body mass was calculated.

Sample size calculations showed that with a sample of around 20 women engaged in active exercises compared to a control group of around three to four times would be adequate to show a difference in the BMD loss in pregnancy of 50 % or more at a significance level of 0.05, with a power of 80 %, based on our previous data on the magnitude and range of BMD loss in pregnancy using a similar research methodology (mean BMD at early pregnancy of around 0.56 mg/cm^3^ and a 5 % loss from early pregnancy to late pregnancy).

Univariate analysis of the data was performed by Chi-square tests for discrete entities and by paired and unpaired Student’s *t* tests for continuous variables where appropriate, with prior testing for normal distribution of the data. Regression models were then constructed using BMD loss in pregnancy as the dependent variable, and parameters found to be statistically significant on univariate analysis were entered into the equation to verify whether exercises were a significant determinant. A *p* value of <0.05 was considered significant in this study. Approval was obtained from the hospital cluster ethics committee for the utilization of quantitative ultrasound for bone density measurement in pregnancy.

## Results

A total of 24 physically active women were recruited. Most were engaged in active exercises or training due to their profession. These included ten professional dancers or professional dance teachers, two tennis coaches, three ice skating coaches, two gymnastics coaches and four athletic/sports/fitness coaches, but there were also two amateur marathon runners and one amateur triathlon runner. The reported level of physical training at the time of recruitment was significantly higher than in later pregnancy (mean 13.1 h, SD 3.33) (median recruitment gestation 17 weeks, range 12–19 weeks), but up to 24–26 weeks, all of them still reported active physical training of over 10 h per week. These were compared to 94 non-exercising low-risk women not actively engaged in physical exercises, and their mean duration of leisure exercises per week was 0.08 h between 24 and 28 weeks (SD 0.28).

Comparison of the changes in weight, body mass index and body fat percentage from early to late gestation showed that both the exercising and non-exercising group showed significant increases in these parameters The mean BMD changes in the non-exercising group showed a typical BMD fall from early gestation to late gestation of around 6.8 %, while the exercising group showed a drop of around 2.4 % (Table 1).

**Table 1:** Changes in anthropometric parameters from early to late pregnancy

	Early pregnancy (<20 weeks)	Late pregnancy (36–40 weeks)	Mean difference (95 % CI)
Weight (kg)			
Exercising group	50.8 (SD 4.99)	60.1 (SD 7.31)	9.3 (7.4 to 10.7)
Non-exercising group	57.7 (SD 8.55)	67.8 (SD 8.56)	10.1 (9.4 to 10.7)
Body mass index (kg/cm^2^)			
Exercising group	21.0 (SD 1.75)	25.7 (SD 2.32)	4.75 (4.29 to 5.21)
Non-exercising group	23.5 (SD 3.23)	27.7 (SD 3.20)	4.13 (3.86 to 4.39)
Body fat composition (%)			
Exercising group	27.4 (SD 3.5)	34.4 (SD 3.11)	6.96 (6.06 to 7.85)
Non-exercising group	31.3 (SD 5.86)	38.8 (SD 5.17)	7.4 (6.8 to 8.0)
Mean BMD (g/cm^2^)			
Exercising group	0.6085 (SD 0.085)	0.5935 (SD 0.09)	−0.015 (−0.0006 to −0.029)
Non-exercising group	0.5963 (SD 0.015)	0.5543 (SD 0.043)	−0.041 (−0.033 to −0.050)

*p* value by paired *t* tests; all *p* values <0.001

*CI* confidence interval

The exercising women were younger (29.2 years versus 31.4 years, *p* = 0.015) and had lower body weight (50.8 kg vs. 57.7 kg, *p* < 0.001), body mass index (BMI; 21 vs. 23.5 kg/cm^2^, *p* < 0.001) and body fat percentage (27.4 vs. 31.3 %, *p* = 0.002) in early pregnancy compared to the non-exercising group, but there was no difference in their early pregnancy BMD values, or their weight and fat gain during pregnancy. A larger proportion of the exercising group were primiparous (83 %) compared to the non-exercising group, but the difference was not statistically significant. A marginal fall in BMD of 0.015 g/cm^2^ (SD 0.034) was demonstrable from early to late gestation in the exercising women, which was significantly lower than that of non-exercising women (0.041 g/cm^2^; SD 0.042; *p* = 0.005) (Table 2). When the early and late pregnancy BMD values of the study group was adjusted by controlling for the differences in age and early pregnancy BMI differences with the control group, the derived pregnancy BMD loss in this group was 0.020 g/cm^2^ (SD 0.038), which remained significantly lower than the corresponding loss in the non-exercising controls (*p* = 0.026) (Table 2).

**Table 2:** Mean anthropometric and BMD changes in those with active physical training during pregnancy and those with no exercises

	Exercising (*n* = 24) (SD)	Non-exercising (*n* = 94)	*p* value	MD (95 % CI)
Age (years)	29.2 (4.03)	31.4 (SD 3.82)	0.015	−2.17 (−3.92 to −0.42)
Parity				
Primiparous	20 (83.3 %)	60 (63.8 %)	0.11	
Multiparous	4 (16.7 %)	34 (36.2 %)		
Exercises per week at early gestation (<20 weeks) (h)	13.1 (3.33)	0.08 (0.28)	<0.001	12.9 (12.3 to 13.6)
Height (cm)	155.4 (4.61)	156.4 (SD 5.6)	0.43	−0.97 (−3.42 to 1.43)
Early pregnancy weight (kg)	50.8 (4.99)	57.7 (8.55)	<0.001	−6.87 (−10.48 to 3.25)
Early pregnancy BMI (kg/cm^2^)	21.0 (1.75)	23.5 (3.24)	<0.001	−2.55 (−3.91 to −1.18)
Early pregnancy body fat composition (%)	27.4 (3.5)	31.3 (5.86)	0.002	−3.92 (−6.4 to −1.45)
Early pregnancy BMD (g/cm^2^)	0.608 (0.085)	0.596 (0.015)	0.22	0.19 (−0.006 to 0.03)
Weight gain in pregnancy (kg)	9.3 (3.45)	10.1 (3.21)	0.28	−0.79 (−2.27 to 0.68)
Body fat accumulation in pregnancy (%)	6.95 (2.11)	7.45 (2.95)	0.43	−0.49 (−1.76 to 0.77)
Total BMD loss in pregnancy (g/cm^2^)	0.015 (0.034) (range −0.04 to 0.06; median 0.016)	0.0419 (0.0421) (range −0.08 to 0.17; median 0.038)	0.005	−0.026 (−0.045 to −0.008)
Adjusted BMD loss in pregnancy (g/cm^2^)^a^	0.0209 (0.038)	0.0419 (0.042)	0.026	−0.021 (−0.039 to −0.002)

*BMI* body mass index, *SD* standard deviation, *MD* mean difference, *CI* confidence interval

^a^Adjusted BMD for exercising women after controlling for BMI and age difference with control group

There were no significant differences in the incidence of common antenatal complications, such as antenatal anemia, gestational diabetes mellitus or hypertensive disorders between the exercising and non-exercising groups. All of the exercising women recruited for the study delivered after 37 weeks, while three women initially included in the non-exercising group delivered before 37 weeks and were excluded in the final analysis. The mean gestation at delivery in the final cohort again did not differ between the two groups. In addition, while the birth weight of the babies of the exercising group were slightly lower than those of the non-exercising group (3,050 vs. 3,250 g) by around 200 g, the difference was not statistically significant. The cesarean delivery rate was also lower in the exercising group as compared to the non-exercising group (8.3 vs. 21.2 %), probably because of more multiparous women in the latter group requiring repeat cesarean section after a previous cesarean. The difference was not statistically significant (Table 3).

**Table 3:** Pregnancy outcome between exercising and non-exercising women

	Exercising (*n* = 24)	Non-exercising (*n* = 94)
Gestation at delivery (weeks)	38.9 (1.70)	39.1 (1.50)
Birth weight (g)	3,050 (280)	3,250 (430)
Antenatal complications		
Antenatal anemia	1 (4.2 %)	6 (6.3 %)
Gestational diabetes mellitus	1 (4.2 %)	7 (7.4 %)
Hypertensive disorders	2 (8.3 %)	5 (5.3 %)
Antepartum hemorrhage	1 (4.2 %)	2 (2.1 %)
Mode of delivery		
Normal spontaneous	20 (83 %)	70 (74.4 %)
Assisted vaginal delivery	2 (8.3 %)	4 (4.2 %)
Cesarean section	2 (8.3 %)	20 (21.2 %)
Low 5-min Apgar score <4	0	1 (1 %)

A logistic regression model constructed using the presence or absence of BMD loss in pregnancy as the dependent variable against the significant factors identified in univariate analysis showed that lower early pregnancy body mass index (*p* = 0.02) increased the risk of BMD loss (OR 1.45), while more hours of exercise in pregnancy (*p* = 0.02) reduced this risk (OR 0.9) (Table 4). A linear regression model using BMD loss in pregnancy as the dependent variable against other significant parameters showed that the hours of exercises in pregnancy remained a significant factor associated with this loss (*p* = 0.003) (Table 5).

**Table 4:** Logistic regression using bone mineral density loss in pregnancy as dependent variable against significant parameters

Variable	B	SE	Wald	Significance	Odds ratio	95 % CI
Age	0.0046	0.0675	0.0047	0.94	1.00	0.88–1.14
Early pregnancy body mass index	0.3756	0.1684	4.974	0.02	1.45	1.04–2.02
Early pregnancy body fat %	−0.1265	0.0841	2.262	0.13	0.88	0.74–1.03
Exercise hours per week	−0.101	0.045	5.041	0.02	0.90	0.82–0.98

*SE* standard error, *CI* confidence interval

**Table 5:** Linear regression using BMD loss in pregnancy against significant parameters

Variable	B	SE	Beta	Significance	95 % CI
Age	0.00016	0.001	0.016	0.86	−0.002 to 0.002
Early pregnancy weight	0.00009	0.001	0.020	0.92	−0.002 to 0.002
Early pregnancy body mass index	0.00197	0.003	0.150	0.48	−0.004 to 0.008
Early pregnancy body fat %	−0.00139	0.001	−0.188	0.18	−0.003 to 0.001
Exercise hours per week	−0.00236	0.001	−0.295	0.003	−0.004 to −0.001

## Discussion

The data presented in this study confirmed a demonstrable progressive fall in BMD at the os calcis as measured by quantitative ultrasound from early to late pregnancy. The mean decrease in BMD was around 6 % of the early pregnancy BMD value (0.0365/0.5988 g/cm^2^). This finding was consistent with similar studies utilizing various means to measure BMD loss in pregnancy [3–5, 7, 8], as well as in a previous cohort that we reported using the same quantitative ultrasound system [6, 16]. During pregnancy, marked enhancement of bone turnover could be shown together with loss in BMD that is believed to be reversible [17]. The cumulative calcium deficit from pregnancy and lactation approaches around 6 % of the total body calcium store [2, 18]. This loss was readily detectable using quantitative ultrasound BMD measurements across different gestations, as the magnitude of measurable loss during pregnancy should exceed the minimal significant measurable differences or the expected precision error of these systems [16].

The data from this study showed that in a selected group of actively exercising women, the normal physiological fall in BMD in the os calcis as measured by quantitative ultrasound was attenuated as compared to non-exercising controls, supporting the hypothesis that exercise during pregnancy could have an impact on bone metabolism. Previous studies evaluating the relationship of exercise with BMD usually focused on pediatric and adolescent age groups [12–14], reproductive age women [15] or postmenopausal age groups [19]. We are yet to find similar evaluations directly in a pregnant cohort in the literature. A previous study on the effects of prolonged bed rest during pregnancy and its effects on bone metabolism showed a significant increase in bone turnover markers in these immobilized women, indicating a negative impact on BMD [20]. These findings apparently supported our hypothesis that exercises would help to attenuate the physiological loss in BMD.

On the other hand, a study in a small cohort of postpartum women showed a lack of significant impact of self-selected recreational exercises on early postpartum lactation-induced BMD loss [21]. However, the level of physical activity described in the study was likely to be lower than in our study, and the interval between assessments was limited to only 3 months. In addition, it would also be difficult to generalize such findings to BMD changes during pregnancy.

The effects of exercise on pregnancy outcome have been extensively studied in the past. In particular, the correlation between exercises and birth weight has been studied using various methods. In a recent study, no significant associations with birth weight was seen in a large cohort undergoing moderate to heavy physical activity in the second and early third trimester [22]. However, the mean activity level of the women in this study was around 3–4 h per week, while the activity level in the cohort in our study was significantly higher. Our data did show a slight difference in birth weight of around 200 g between the exercising and non-exercising groups, though this difference did not reach statistical significance. Nevertheless, we estimated that a significant difference would have been observed if our cohort were much larger. Taking into consideration the high physical activity level of the women in our study, it would be reasonable to consider them to be at high risk, similar to those engaged in “elite” or “competitive” sports, and institute appropriate fetal surveillance [23].

The small sample size in this study was unable to verify the potential benefits of exercise during pregnancy in reducing the risk of gestational diabetes mellitus or pre-eclampsia [24, 25], or the relationship to preterm delivery. It is of interest to note that none of the recruited women in the exercise group had preterm delivery before 37 weeks, while three from the non-exercising arm had preterm delivery precluding completion of the second BMD assessment and was thus excluded from the final analysis. Thus, in line with the findings of population-based data [26], the risk of preterm delivery was apparently not increased in our cohort and could possibly be reduced.

Our study was limited by the small number of exercising women that we could recruit. On the other hand, as over two-thirds of these recruited subjects were engaged in physical activity via their occupation rather than as leisure time activity, it could be argued that their activity levels were constant and regular and also explained why the activities extended well into the third trimester for many of them. This picture was quite different from other studies that described women involved in strenuous jobs were more likely not to work at all during the third trimester compared to those in less physically demanding jobs, or that they would change to something less intensive [27]. We were unable to fully match the age and BMI of the controls with the study subjects, as consecutive non-exercising women booked after the index cases were recruited as controls to avoid selection bias. Nevertheless, it could be seen that the actual age difference between the two groups (29.42 vs. 31.4 years) was small and was unlikely to bias the BMD values. While controlling for BMI would probably provide a more precise comparison, this was in practice difficult to achieve. As observed in our data, the exercising women naturally had lower BMI at all stages of pregnancy as compared to the controls, so that we believe it should be justifiable to compare the crude BMD differences between the two groups. In fact, when we attempted to derive an adjusted BMD value for early and late pregnancy for the exercising women using BMI and age as the confounding variables against the controls as the standard, while the BMD loss in pregnancy in the exercising women became exaggerated, this value still remained significantly lower than that of the controls, indicating a genuine difference between the two groups.

Another limitation lies in the fact that we were unable to gauge the levels of physical exertion of the subjects using more scientific measurements such as oxygen consumption or maximal heart rate, and could only rely on their history and the reported duration that they engaged in these physical exercises. Thus, the heterogeneity of their physical activity as well as the varying intensity levels could have attenuated the differences from the control non-exercising group. Despite such possibilities for bias against finding any significant differences, our results were still in support of the hypothesis that intensive physical exercises of weight-bearing type in pregnancy could reduce bone loss during pregnancy. Theoretically, the ideal design for a study of this nature would be to recruit a cohort of women with identical exercise levels in early pregnancy, and then randomize them either to undergo intensive exercises in pregnancy or no exercises. In practice, such allocations would not be feasible as it would be most unlikely that allocated subjects would be able to comply with the prescribed intensive exercise regimes, particularly if they were not used to the level of physical exertion before or at early pregnancy. Thus, the settings presented in this study remained the only practical comparison to evaluate the effects of exercises in BMD changes during pregnancy.

The long-term effects of continuing exercises into pregnancy and beyond a regular level have recently been studied. Women who voluntarily maintained their exercise regimen during pregnancy were studied 18 months to 2 years after their index pregnancy. These women were found to continue to exercise over time at a higher level than those who stopped during pregnancy, and were able to maintain their long-term fitness and to have a low cardiovascular risk profile in the peri-menopausal period [28]. However, whether the continuation of exercises into pregnancy and beyond would have benefits on BMD in later life remains to be evaluated.

In summary, while our data showed preliminary evidence that exercises during pregnancy would contribute benefits to maintaining BMD, larger-scale studies involving more sophisticated and precise measurements of the level of exercises in pregnancy, and refining to a cohort with more homogenous physical activities, could provide more information on the physiology and mechanisms relating the benefits of exercises to bone loss in pregnancy. The long-term benefits of exercises in pregnancy on later life osteoporosis risks would also need further exploration.
